# Switchable friction enabled by nanoscale self-assembly on graphene

**DOI:** 10.1038/ncomms10745

**Published:** 2016-02-23

**Authors:** Patrick Gallagher, Menyoung Lee, Francois Amet, Petro Maksymovych, Jun Wang, Shuopei Wang, Xiaobo Lu, Guangyu Zhang, Kenji Watanabe, Takashi Taniguchi, David Goldhaber-Gordon

**Affiliations:** 1 Department of Physics, Stanford University, Stanford, California 94305, USA; 2 Department of Physics, Duke University, Durham, North Carolina 27708, USA; 3 Department of Physics and Astronomy, Appalachian State University, Boone, North Carolina 28608, USA; 4 Center for Nanophase Materials Sciences, Oak Ridge National Laboratory, Oak Ridge, Tennessee 37831, USA; 5 Institute of Physics, Chinese Academy of Sciences, Beijing 100190, China; 6 National Institute for Materials Science, 1-1 Namiki, Tsukuba 305-0044, Japan

## Abstract

Graphene monolayers are known to display domains of anisotropic friction with twofold symmetry and anisotropy exceeding 200%. This anisotropy has been thought to originate from periodic nanoscale ripples in the graphene sheet, which enhance puckering around a sliding asperity to a degree determined by the sliding direction. Here we demonstrate that these frictional domains derive not from structural features in the graphene but from self-assembly of environmental adsorbates into a highly regular superlattice of stripes with period 4–6 nm. The stripes and resulting frictional domains appear on monolayer and multilayer graphene on a variety of substrates, as well as on exfoliated flakes of hexagonal boron nitride. We show that the stripe-superlattices can be reproducibly and reversibly manipulated with submicrometre precision using a scanning probe microscope, allowing us to create arbitrary arrangements of frictional domains within a single flake. Our results suggest a revised understanding of the anisotropic friction observed on graphene and bulk graphite in terms of adsorbates.

## Abstract (web summary)

Graphene can exhibit pronounced frictional anisotropy, which was thought to arise because of nanoscale ripples. Here, the authors provide evidence that this effect could instead be a result of adsorbates that self-assemble into a highly regular superlattice of stripes with a period of four to six nanometres.

## Graphical abstract

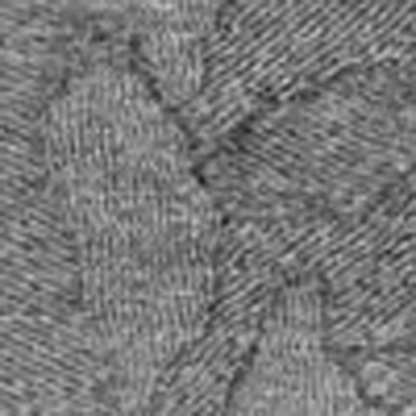

Nanometre-scale surface textures with long-range order often give rise to pronounced frictional anisotropy. These textures sometimes originate from crystal structures: periodic tetrahedral reversals in the antigorite lattice create nanoscale surface corrugations, which generate the anisotropic friction that governs certain seismic processes^1^. A large frictional anisotropy similarly arises for some quasicrystal intermetallics, whose surfaces are textured by atomic columns^2^. Rotationally aligned molecules also form ordered nanotextures with associated anisotropic friction, as observed in organic crystals^3^. In adsorbed organic films^4^,^5^,^6^, rotational symmetry of the host surface permits multiple stable molecular orientations, yielding frictional domains with anisotropy along different axes.

From a technological standpoint, nanometre-scale systems with such multistability are appealing platforms for switches or memories. Bistable states in redox centres^7^, rotaxane molecules^8^ and iron clusters^9^ can be addressed and switched using scanned probes, enabling dense information storage. Multistable nanotextures could find application in nanoelectromechanical systems if the friction-producing textures could be dynamically controlled, as in biomimetic tapes with magnetically actuated micropillars^10^. Existing schemes for tuning friction at submicrometre scales include Fermi level modulation in silicon^11^ and mechanical oscillation of a sliding contact^12^—nonhysteretic techniques, which require maintenance of a voltage or oscillation, a disadvantage for circuitry.

In this study, we identify a friction-producing nanotexture that naturally forms on graphene exposed to laboratory air and exploit its multistability to hysteretically switch friction with submicrometre precision. Using high-resolution atomic force microscopy (AFM), we directly image a superlattice of nanoscale stripes on exfoliated graphene and we show that this striped nanotexture produces the anisotropic friction previously observed^13^,^14^,^15^ on graphene monolayers. This nanotexture strongly resembles patterns of adsorbates observed on graphite^16^,^17^,^18^ and we induce an apparently identical nanotexture on flakes of hexagonal boron nitride (hBN) using a thermal cycling procedure. Consistent with the adsorbate picture, we can rapidly and predictably reorient the frictional domains by scanning a probe tip along the flake in a chosen direction—a departure from nanoassembly techniques^19^ such as dip-pen nanolithography^20^ and nanografting^21^, for which writing a different ‘colour' requires submerging the sample in a different ‘ink'.

## Results

### Superlattice of nanoscale stripes

**Figure 1:**
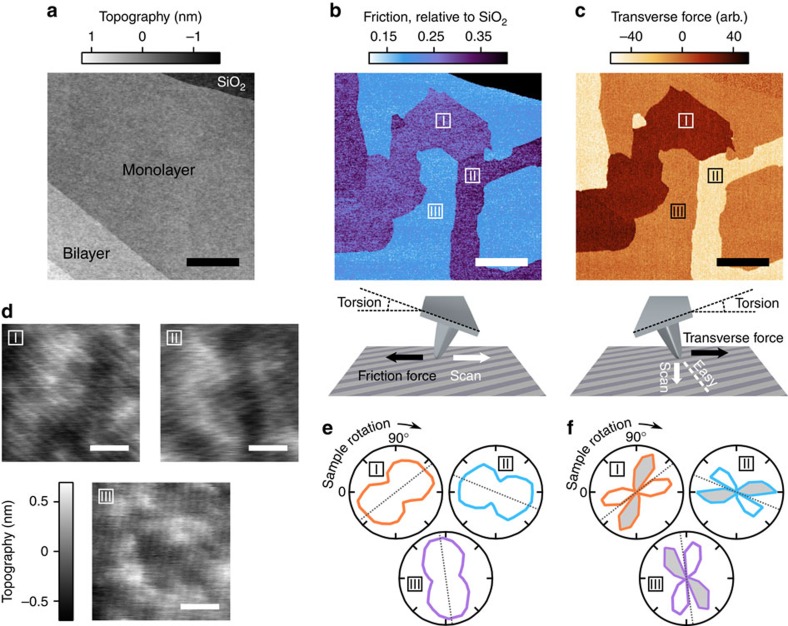
Stripes on exfoliated graphene. (**a**) Contact mode topography scan of a graphene flake on silicon oxide, showing monolayer, bilayer and trilayer regions. Scale bar, 3 μm. (**b**) Simultaneously recorded friction signal (upper panel), showing three distinct domains of friction labeled I, II and III. Lower panel: cartoon of the friction imaging mode. The cantilever is scanned laterally and friction between the tip and sample produces the measured torsion of the cantilever. (**c**) Transverse force signal (upper panel) from the same region as in **b**, measured by recording the torsion while scanning the cantilever longitudinally (lower panel). Surface anisotropy pushes the tip towards the local ‘easy' axis, creating a transverse force that twists the cantilever. (**d**) Tapping mode topography scans of the graphene monolayer, taken within each of the three domains. Each domain is characterized by stripes of period 4.3±0.2 nm along one of three distinct axes rotationally separated by 60°. Scale bars, 20 nm. (**e**) Friction relative to SiO_2_ for each domain as a function of clockwise sample rotation angle; zero degrees corresponds to the orientation shown in **a**–**c**. For each polar plot, the origin and circumference correspond to relative friction values of 0.15 and 0.4, respectively. Dotted lines indicate the sample rotations at which the stripes shown in **d** are parallel to the scan axis. The friction signal is approximately sinusoidal, with the highest friction produced when stripes are parallel to the scan axis. (**f**) Transverse force signal for each domain as a function of clockwise sample rotation angle. Unshaded and grey-shaded regions indicate positive and negative transverse signals, respectively. The origin of each polar plot is zero. The transverse signal for a given domain switches sign as the stripe axis rotates through the lateral axis.

To image friction, we measure the deflection (diving board motion) and torsion (axial twist) of a scanned AFM cantilever in light contact with the sample. The deflection signal primarily contains topographic information, while the meaning of the torsion signal depends on scan direction. For lateral scanning (motion perpendicular to cantilever axis; Fig. 1b lower panel), the torsion measures lateral tip-sample forces commonly interpreted as friction forces. In this ‘friction-imaging' mode, tip-sample forces transverse to the scan direction result in deflection, contributing spurious topographic signals. When the cantilever is scanned longitudinally (Fig. 1c lower panel), the torsion signal directly measures tip-sample forces transverse to the scan direction. For an isotropic surface, this ‘transverse force' signal is zero.

As reported previously^13^,^14^, the friction signal of exfoliated monolayer graphene flakes on silicon dioxide reveals up to three distinct domains of friction despite a featureless topography signal (Fig. 1a,b). The domains vary in size from tens of nanometres to tens of micrometres and produce sharp contrast in transverse force, confirming their anisotropic character (Fig. 1c). Tapping-mode AFM images taken with ultrasharp tips within the different domains (Fig. 1d) reveal periodic stripes along axes rotationally separated by 60° (angular orientation does not measurably vary within a given domain; see Supplementary Note 1). Within experimental error (typically ±0.2 nm), stripe period (typically ∼4 nm) does not change across a sample, although we have observed global changes in stripe period after thermal cycling (for example, from 4 to 6 nm; see Supplementary Note 2). Peak-to-trough stripe amplitude ranges between 10 and 100 pm, but strongly depends on tip conditions and oscillation parameters.

The observed frictional anisotropy of a given domain respects the symmetry of the stripe-superlattice. The friction signal approximately tracks the cosine of the angle between scan axis and stripes (Fig. 1e)—friction is maximized when the two are aligned—whereas the transverse force is zero when the stripes are perpendicular or parallel to the scan axis, as required by symmetry (Fig. 1f). In between these zeros, the transverse force changes sign so as to guide the sliding tip towards the low friction axis (lower panel in Fig. 1c). We conclude that the stripes in graphene produce the observed friction anisotropy, similar to friction-producing nanotextures in other systems^1^,^2^,^3^,^4^,^5^,^6^.

**Figure 2:**
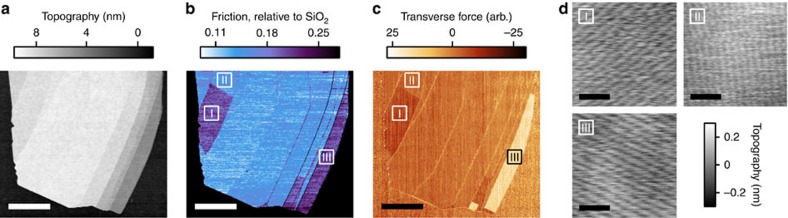
Stripes on exfoliated hBN. (**a**) Contact mode topography scan of a terraced hBN flake, thickness 5–9 nm, after thermal cycling in liquid nitrogen. Scale bar, 5 μm. (**b**,**c**) Simultaneously recorded friction signal (**b**) and separately recorded transverse force signal (**c**) showing the presence of three distinct domains (I, II and III). The contrast between I and III is weak in friction, but strong in transverse force. (**d**) Tapping mode topography scans of the three domains, taken in the regions indicated in **b** and **c**. Each domain is characterized by stripes of period 4.7±0.2 nm along one of three distinct axes rotationally separated by 60°. Scale bars, 20 nm.

The stripes are not unique to monolayer graphene on SiO_2_. We observe stripe domains and anisotropic friction on graphene flakes up to 50 nm thick (the maximum thickness investigated) without change in stripe period and with minor change in magnitude of frictional anisotropy (Supplementary Note 3), as well as on graphene flakes on different substrates (Supplementary Note 4). Stripe domains can also form on exfoliated flakes of hBN on SiO_2_: single crystals show at most three distinct domains of anisotropic friction (Fig. 2b,c), each characterized by a different orientation of stripes, whose typical period is ∼4 nm (Fig. 2d). As for graphene, the friction signal is maximized when scanning along the stripes. However, whereas we observed stripes on nearly all graphene flakes as exfoliated, only occasionally did we observe stripes on hBN as exfoliated. We found that a cryogenic thermal cycle such as immersion in liquid nitrogen and subsequent removal to ambient conditions (Methods) would reliably produce stripes on hBN. A full understanding of the effect of thermal cycling is beyond the scope of our study; our limited variable-temperature AFM experiments found stripes to form on hBN on cooling from 300 to 250 K, although vacuum conditions probably influenced the evolution with temperature (Supplementary Note 5).

**Figure 3:**
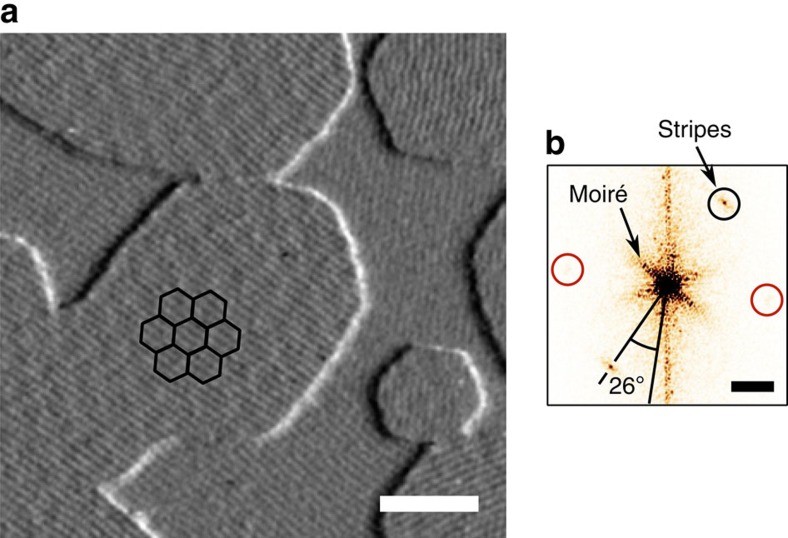
Orientation of stripes on graphene and hBN. (**a**) Tapping mode topography image of graphene islands grown by van der Waals epitaxy on exfoliated hBN. The image has been differentiated along the horizontal axis for clarity. Graphene islands can be distinguished from the hBN surface by the presence of a moiré pattern, which is partially outlined in black for one of the grains. The sample surface is covered with stripes of period 4.3±0.1 nm, oriented along one of three distinct axes rotationally separated by 60°. The stripe period is the same on graphene and hBN, and the stripes frequently appear to cross the graphene/hBN boundary without a phase slip. Scale bar, 50 nm. (**b**) Fast Fourier transform (FFT) of the topography signal used to produce **a**. The moiré pattern within the graphene grains appears as a sixfold-symmetric pattern with segments extending ∼70 μm^−1^ from the origin; these protruding segments are parallel to the momentum-space moiré lattice vectors. The dominant stripe domain on graphene and hBN produces a pair of isolated points in the FFT, one of which is circled in black. The stripe axis is rotated 26±4° from the moiré lattice vectors, indicating that the stripe axes are nearly aligned with the armchair axes of the graphene and hBN. The quoted angular precision reflects the width of the moiré peaks; we also expect a few-degree systematic error in the angular estimate, as a misalignment between graphene and hBN lattices of 0.1°—a reasonable expectation for van der Waals epitaxial heterostructures^23^—would rotate the moiré pattern by 4° with respect to the graphene lattice. The small area of nearly vertical stripes in **a** produces a pair of points, circled in red, which can barely be seen with this colourscale. Scale bar, 100 μm^−1^.

The behaviour of stripes on epitaxial heterostructures of graphene and hBN implies that stripes on both materials share a common origin. The nearly perfect rotational alignment between stacked lattices^22^ results in a moiré pattern with lattice constant ∼14 nm in regions where graphene has grown on the hBN (Fig. 3a). Despite this additional superstructure, stripes form on exposed layers of both graphene and hBN with no measurable difference in period and often appear to maintain phase across a graphene/hBN boundary. Furthermore, using the moiré pattern to infer lattice orientation^23^ (Fig. 3b), we find that the stripes run along the armchair axes of both graphene and hBN in all 25 epitaxial heterostructures and 5 mechanically assembled heterostructures that we studied (Supplementary Note 6).

Previous studies have ascribed the anisotropic friction in monolayer graphene to periodic ripples in the graphene sheet induced by stress from the substrate^13^,^14^,^15^. Although our data confirm the presence of periodic structure, the extreme similarity of the stripes on graphene and hBN—materials with different bending stiffness and response to stress^24^—suggests that the stripes are adsorbates rather than features of the crystals themselves. The orientation of the stripes further rules out periodic ripples, which are expected to produce a high friction axis perpendicular to the stripes^13^,^14^ and a zigzag stripe axis^15^,^25^—both opposite to our findings. Periodic ripples have never been observed in scanning tunnelling microscopy (STM) of the graphene lattice and our STM data are no exception (Supplementary Note 7); on the other hand, adsorbates can be disturbed by the pressure of the STM tip under standard imaging conditions^26^, perhaps explaining why the stripes that we observe have not previously been reported.

Various organic adsorbates are known to self-assemble into nanoscale stripes on graphite. Surfactant molecules, for instance, form stripes^17^ whose 4–7 nm period is set by molecular length and Debye screening^27^; anisotropic van der Waals interactions align the stripes along the armchair axes^17^. Alkanes also produce armchair-aligned stripes of 4 nm period on graphite, where the period is again determined largely by molecular length^16^. Self-assembly of inorganic species has been reported as well: Lu *et al*.^18^,^28^ observed crystallographically aligned stripes of 4 nm period on graphite submerged in water and correlated the stripes with the presence of dissolved nitrogen gas. Noting that gas enrichment at the interface between a hydrophobic surface and water is theoretically expected^29^, Lu *et al*.^18^,^28^ argued that the stripes were self-assembled columns of molecular nitrogen adsorbed to the graphite surface. Stripes of similar period were later observed in ambient on multilayer epitaxial graphene^30^,^31^; following Lu *et al*.^18^,^28^, these stripes were attributed to nitrogen adsorbates trapped at the interface between graphene and an ambient water layer. We note that these studies^18^,^28^,^30^,^31^ do not provide a direct chemical analysis of the stripes to prove their nitrogen content. Why stripes should form instead of a homogeneous layer of nitrogen is also unexplained.

We propose that the stripes on graphene and hBN are self-assembled environmental adsorbates, in view of their similarity to stripes formed by adsorbates on graphitic surfaces^16^,^17^,^18^,^27^,^28^,^30^,^31^ and their aforementioned dissimilarity to structural ripples, as well as our ability to manipulate the stripes by physical contact (see below). Although direct determination of the chemical makeup of the stripes is beyond the scope of our work, our data suggest that the species that self-assembles is airborne and ubiquitous in the laboratory, as stripes of uniform period fully cover our cleaved or annealed crystal surfaces that have not been exposed to any chemical processing (Methods). From this perspective, an interpretation in terms of nitrogen and water^18^,^28^,^30^,^31^ or other common inorganic molecules is attractive. However, hydrocarbons are also plentiful in laboratory air (arising from, for instance, outgassing plastics or vacuum pump oil) and certain species could preferentially attach to graphene or hBN due to lattice match^16^.

Recent work resolved nanoscale stripes in the transverse force response of bulk graphite and ascribed them to a novel puckering-induced stick–slip friction process^32^. These stripes produced domains of anisotropic friction^33^ such as those on graphene and hBN. We suggest a reinterpretation of these data in terms of adsorbates, which would unify our understanding of anisotropic friction in graphite, graphene and hBN.

### Manipulation of frictional domains

**Figure 4:**
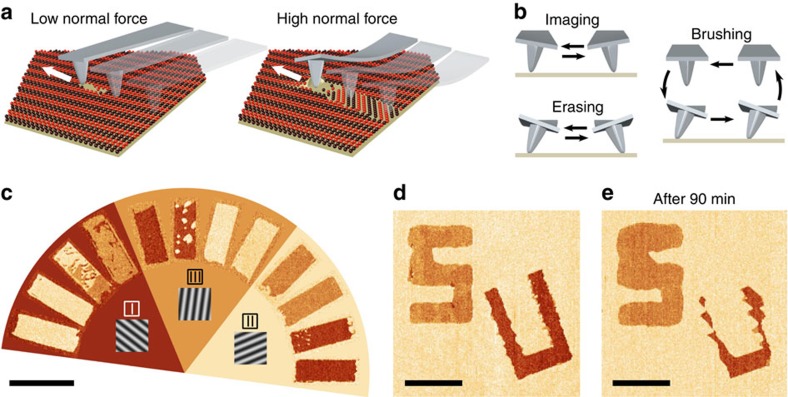
Rewritable friction on monolayer graphene. (**a**) Cartoon illustrating the response of the striped adsorbates to the scanning tip. At low normal force, the tip minimally disturbs the stripes as it scans the surface and the stripe structure rapidly heals. At high normal force, the stripe structure is heavily disturbed, creating a new stripe domain in the wake of the scanning tip. (**b**) Summary of our scanning modes. For imaging, we rapidly scan the cantilever back and forth at low normal force, while slowly moving it in the direction perpendicular to the fast scan axis. The erasing mode is identical, but at high normal force. For a brush stroke, we raster-scan the cantilever such that the tip only moves in one direction when in contact with the sample. After scanning each line, we lift the cantilever, move it to the start of the next line and touch down again. (**c**) Domain switching as a function of scan angle on the monolayer flake studied in Fig. 1, rotated as in Fig. 1a–c. The image shown is a collage of 12 transverse force images, each taken after executing a single 3 μm by 1 μm brush stroke on a canvas composed initially of a single domain. For each canvas domain we show four brush strokes nearly parallel with the canvas stripes, where each brush stroke is directed radially outward from the origin of the semicircle. The brush strokes steer the canvas domain towards the domain whose stripes are next nearest the brush axis. Scale bar, 3 μm. (**d**) Transverse force image immediately after writing block letters ‘S' and ‘U' in domains III and I, respectively, on a canvas of domain II (same flake and orientation as in **a**). The block letter ‘S' was written by ‘erasing', whereas the ‘U' was written using brush strokes. Scale bar, 3 μm. (**e**) Transverse force image of the same area, taken 90 min later. The ‘S' (domain III) has expanded into the canvas, while the ‘U' (domain I) has decayed.

Adsorbates can sometimes be mechanically manipulated by AFM^19^, raising the possibility of patterning friction on these materials. For monolayer graphene on SiO_2_, scanning at the low normal force used for imaging (1 nN) often minimally affects the frictional domains, but scanning at high normal force (30 nN) reproducibly reorients the domains (Fig. 4a). We devised two standard approaches for domain manipulation (Fig. 4b). The ‘brush stroke' consists of raster scanning a rectangular window at high normal force; we retract the tip after every line so that it only scans the sample in one direction. Brush strokes produce reproducible results—often a domain flop—that depend on the scan angle and the initial ‘canvas' domain. For scan angles near the canvas stripe axis, the canvas switches to the domain with stripes next closest to the scan axis (Fig. 4c). Our second approach is to ‘erase' the canvas domain within a rectangular scan window by rapid, back-and-forth scanning at high normal force. This mode destabilizes the domains within the scan window, leaving only the most stable domain, determined primarily by local strain and partly by scan axis. Although strain gradually varies across the flake (see discussion below), erasing still produces deterministic results within a specific region.

The brush stroke and eraser allow us to rapidly create patterns of friction with submicrometre precision. Without optimizing our procedure, creating a block letter ‘S' 5 μm tall using the eraser took 16 min, whereas creating a ‘U' using brush strokes took 36 min (Fig. 4d and Supplementary Movie 1). After writing, the pattern gradually decayed: here the ‘S' widened, while the ‘U' narrowed (Fig. 4e). We wrote the same pattern in different parts of the flake and found that whether a domain grew or shrank with time, and how rapidly it evolved, depended on position. The absence of other obvious symmetry-breaking mechanisms suggests that local strain induced by the substrate determines the relative stability of the domains. Domain stability in turn determines the effective resolution of our patterning technique: although we can write crisp lines 100 nm wide in some parts of a flake, in other parts these features only persist for minutes before decaying to match the canvas domain. In addition, although we can pattern friction on several different monolayer graphene flakes, others show only weak response to both patterning modes described; the strain field in these flakes probably strongly favours the local canvas domain, making the canvas difficult to switch.

Whether patterning friction is possible on thicker crystals requires further investigation. Our first attempts indicate that domains can be rewritten with the eraser or brush stroke, although the resulting domains are not as sharp as on monolayer graphene. Proximity to the substrate could be stabilizing the stripes, allowing for more flexible control of domain shape. Our work underscores the major role played by adsorbates, rather than structural deformation, in determining friction on graphene and hBN—and perhaps on other layered materials, such as transition metal dichalcogenides. The periodic perturbation from the adsorbates might open gaps at the superlattice energy or modify the Fermi velocity in graphene^34^, with measurable consequences for electronic properties of ultraclean graphene/hBN heterostructures^35^.

## Methods

### Sample preparation

Flakes of graphene and hBN were prepared by mechanical exfoliation (3M Scotch 600 Transparent Tape or 3M Scotch 810 Magic Tape) under ambient conditions (40–60% relative humidity) on *n*-doped silicon wafers with 90 or 300 nm of thermal oxide. The substrates were not exposed to any chemical processing following thermal oxidation. For graphene exfoliation, we used bulk crystals of both Kish graphite (Sedgetech, USA) and highly oriented pyrolitic graphite (HOPG ZYA, SPI Supplies, USA) and observed no differences in superlattice phenomena between samples produced using different graphite sources or tapes. For hBN exfoliation, we used bulk crystals provided by Kenji Watanabe and Takashi Taniguchi. We also prepared graphene flakes on other substrates (Supplementary Note 4), including SU-8 epoxy (MicroChem, USA), 200 nm of Au(111) on mica (Phasis, Switzerland) and 5 nm of Pt (electron-beam evaporation) on magnesium oxide (MTI, USA).

We prepared epitaxial graphene heterostructures on oxidized silicon substrates by mechanical exfoliation of hBN followed by graphene growth at 500 °C by a remote plasma-enhanced chemical vapour deposition process described previously^22^. We also mechanically assembled heterostructures of graphene on hBN using both wet^36^ and dry^37^ transfer methods. Polymer residues from the assembly process were removed by annealing samples in a tube furnace for 4 h at 500 °C under continuous flow of oxygen (50 sccm) and argon (500 sccm); before removal to air, we allowed the samples to cool (5–10 °C min^−1^), to below 100 °C under the same flow of oxygen and argon.

### Thermal cycling

We found stripes to appear on our samples after thermal cycling to liquid nitrogen temperatures or below using a variety of methods. Most commonly, and specifically for the sample shown in Fig. 2, we immersed the sample in liquid nitrogen for 1–5 min and then removed it to atmosphere, and blew off the condensation with dry air. This procedure would almost always produce stripes on graphene, hBN or graphene/hBN heterostructures. In other cases, we loaded the sample in the vacuum chamber of a cryostat—either a cryogen-free dilution refrigerator or a Quantum Design PPMS—and thermal cycled to a base temperature between 25 mK and 100 K. Cooling and warming rates varied between 1 and 30 K min^−1^. We warmed up the samples under various atmospheres including moderate vacuum, helium gas or nitrogen gas; in all of these cases (over ten different samples cycled in the dilution refrigerator or PPMS) we found stripes on every flake or heterostructure (totaling several tens) that we checked.

The epitaxial heterostructure in Fig. 3 was not cycled to low temperature: the sample displayed stripes in AFM with no further processing following removal from the growth furnace. Some of our assembled heterostructures (Supplementary Note 6) required a low-temperature thermal cycle to produce stripes after the oxygen/argon anneal, although in other cases we observed stripes without cryogenic treatment.

### AFM and STM measurements

All images shown in Figs 1, 2, 3, 4 were taken with a Park XE-100 AFM under ambient conditions (40–60% relative humidity) except for Fig. 4d,e, which were taken in 10% relative humidity by flooding the chamber of the XE-100 with dry air. (We observed no significant difference in domain mutability or evolution between 10 and 50% relative humidity.) To resolve the stripes in tapping mode, we used sharp silicon probes (MikroMasch Hi'Res-C15/Cr-Au) with a nominal tip radius of 1 nm, a typical resonant frequency of 265 kHz and a typical cantilever *Q* of 400. See Supplementary Note 8 for a detailed interpretation of the tapping mode topography signal.

For measurements in contact mode, we used silicon probes (MikroMasch HQ:NSC19/Al BS-15) with a nominal tip radius of 8 nm and a typical resonant frequency of 65 kHz. We used a normal force setpoint of 1 nN for all friction and transverse force imaging scans shown, with scan rates ∼10 μm s^−1^. See Supplementary Note 8 for a discussion of the friction imaging mechanism. For domain manipulation we used a normal force setpoint of 30 nN. For brush strokes we used scan rates ∼30 μm s^−1^, whereas for erasing we used scan rates ∼300 μm s^−1^.

When imaging friction or transverse force, we collected torsion data for both forward-moving and backward-moving scans. To eliminate offsets in the friction and transverse force signals for Fig. 1e,f, we subtracted backward images from forward images and divided by two. All friction or transverse force images shown are just the forward scan, with any torsion offset eliminated by subtracting the average of forward and backward torsion values on SiO_2_.

To study stripe formation with changing temperature (Supplementary Note 5), we used an Omicron varible-temperature AFM/STM operating in ultrahigh vacuum (UHV; 8 × 10^−11^ mbar). Samples were not baked in UHV before experiments. The sample stage was cooled by a copper braid attached to a cold sink held at low temperature by continuous flow of liquid nitrogen; by this method, we achieved a base temperature of 110 K. We used the same sharp probes as for ambient AFM (MikroMasch Hi'Res-C15/Cr-Au). In UHV, the cantilever *Q* reached 5,000, which significantly restricted scan speed for tapping mode; we therefore used on-resonance frequency-modulation mode, imaging at a typical frequency shift of −30 Hz. For all images, we applied a DC tip-sample bias to nullify the contact potential difference.

STM measurements (Supplementary Note 7) were carried out under ambient conditions using the Park XE-100. We prepared our tip by mechanically cutting a Pt/Ir wire and scanning the sample at high bias voltages until we achieved atomic resolution of the graphene lattice.

### Error bars and lateral calibration

All values quoted for moiré period and angular orientation are extracted from the fast Fourier transform of the AFM images. AFM images of all heterostructures described in this study are corrected for thermal drift by performing an affine transformation to produce regular moiré hexagons (we used the free software Gwyddion, available at gwyddion.net). All error bars reflect the full width at half maximum of the peaks in the fast Fourier transform; for instance, 12.0±0.5 nm means that the full width at half maximum of the peak maps to 1 nm in real space. The lateral scale of the Park XE-100 was calibrated by measuring the moiré period of graphene/hBN heterostructures grown by van der Waals epitaxy, in which the graphene and hBN lattices are nearly perfectly aligned, and defining this period (averaged over several samples) to be 13.6 nm. This definition corresponds to the assumption made in Supplementary Note 6 that the lattice constants for hBN and graphene are *a*_hBN_=0.25 nm and *a*_graphene_=*a*_hBN_/1.018. The lateral scale of the Omicron variable-temperature AFM was calibrated to the lateral scale of the Park XE-100 by measuring the moiré pattern of the same sample in both systems.

## Additional information

**How to cite this article:** Gallagher, P. *et al*. Switchable friction enabled by nanoscale self-assembly on graphene. *Nat. Commun.* 7:10745 doi: 10.1038/ncomms10745 (2016).

## Supplementary Material

Supplementary Information Supplementary Figures 1-7, Supplementary Notes 1-8 and Supplementary References.

Supplementary Movie 1 Patterning frictional domains on monolayer graphene.
