# Supplementary material for: Switchable friction enabled by nanoscale self-assembly on graphene
Source: Nat Commun. 2016 Feb 23;7:10745. doi: 10.1038/ncomms10745 (PMC4766409; doi:10.1038/ncomms10745)
Supplement: Supplementary Information — Supplementary Figures 1-7, Supplementary Notes 1-8 and Supplementary References. [file ncomms10745-s1.pdf]

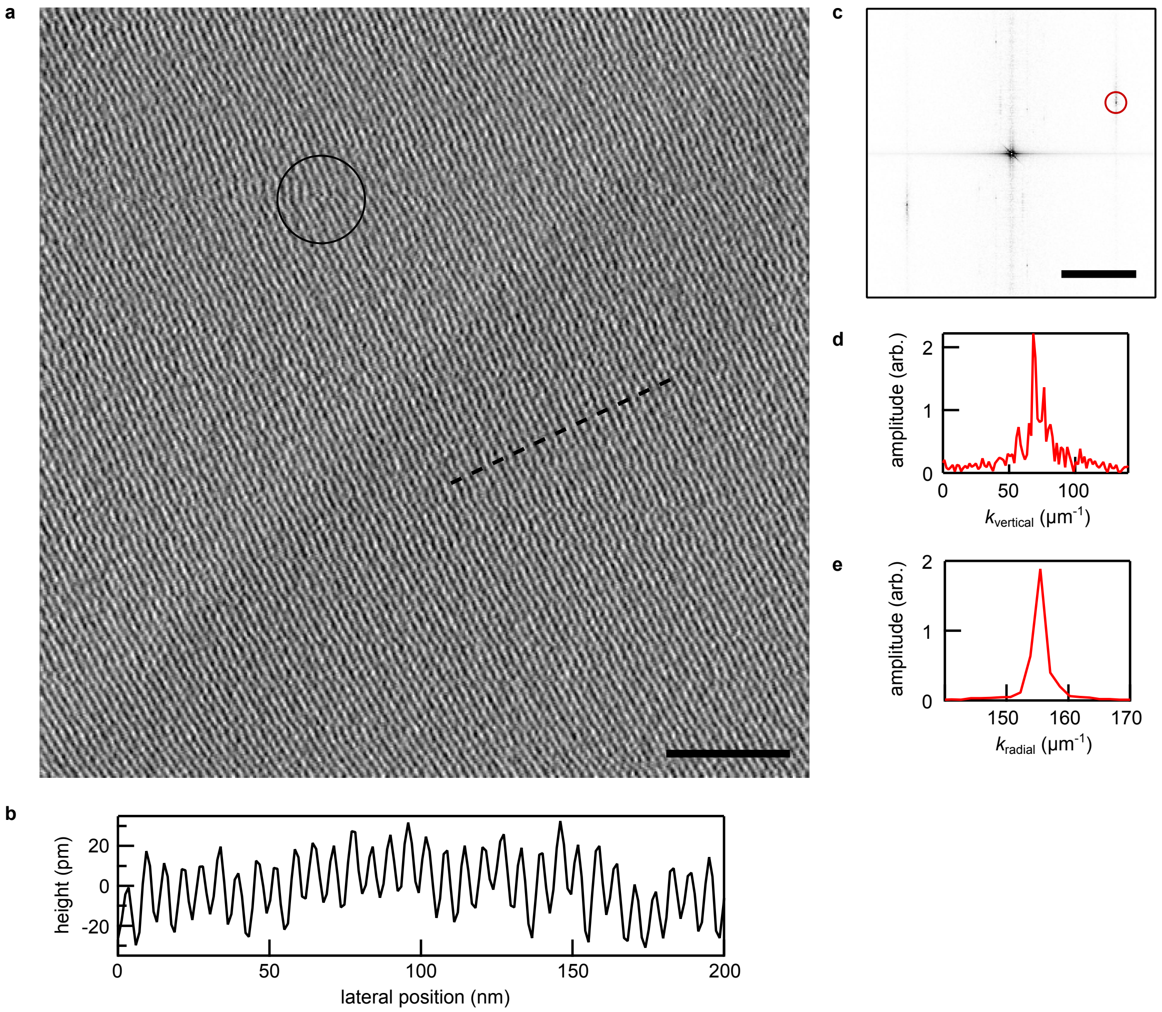

Supplementary Figure 1: Uniformity of stripe-superlattice within a single domain on hBN. **a**, Tapping-mode topography signal, differentiated along the scan axis (horizontal) to suppress the gentle topographic background (the broad feature running bottom left to top right is a smooth depression ∼100 pm deep). Stripe axis runs upper left to lower right. Black circle is centered around a phase slip: three parallel stripes merge into two. Scale bar: 100 nm. **b**, Height (undifferentiated) along black dashed line in **a**. Each point is averaged over 16 nm transverse to the black dashed line. **c**, FFT of the topography signal used to produce **a**. One of the two superlattice peaks is circled in red. Scanner drift smears the peaks along the vertical axis. Scale bar: 100 μm^{-1}. **d**, Vertical cut through the superlattice peak in **c**; $k_{\mathrm{vertical}} = 0$ corresponds to the vertical center of panel **c**. FWHM is 10 μm^{-1}, which corresponds to a 4° error in estimation of the stripe axis. **e**, Radial cut through the superlattice peak in **c**; $k_{\mathrm{radial}} = 0$ corresponds to the center of panel **c**. The stripe period is 6.43 ± 0.04 nm, where the error represents the FWHM of the peak (2 μm^{-1}).

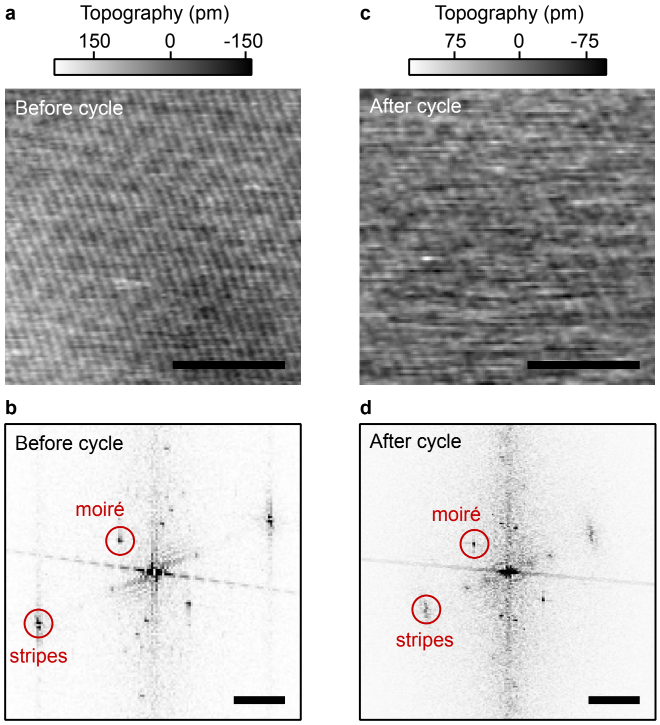

Supplementary Figure 2: Change in global stripe period after thermal cycling a sample. **a**, Topography signal and **b**, fast Fourier transform (FFT) of a mechanically assembled graphene/hBN heterostructure. Stripes of period 4.3 ± 0.1 nm are superimposed on a moiré pattern of lattice constant 11.9 ± 0.4 nm. **c**, Topography signal and **d**, FFT of the same sample after several thermal cycles between 10 K and 390 K in a cryostat (PPMS). The moiré lattice constant has not changed, as expected, but the stripe period is now 5.9 ± 0.3 nm throughout the sample. Topography scale bars: 50 nm. FFT scale bars: 100 μm^{-1}.

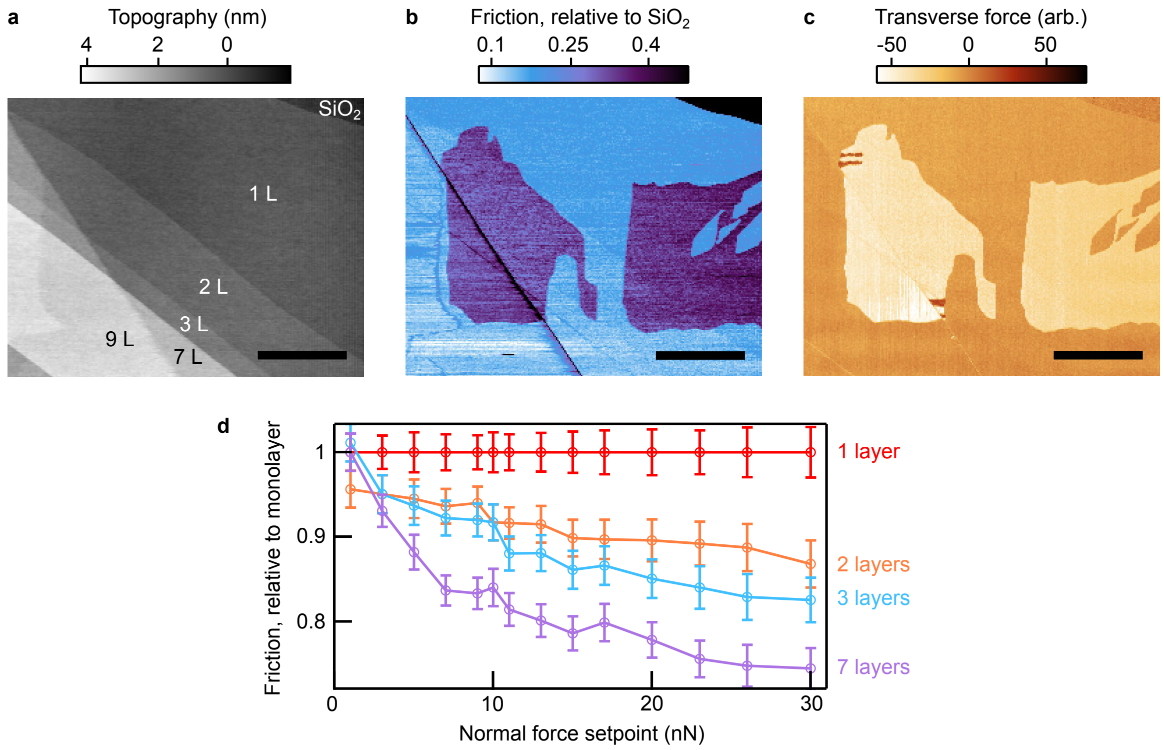

Supplementary Figure 3: Anisotropic friction on a terraced graphene flake. **a**, Contact mode topography signal from a different region of the terraced flake shown in Fig. 1, with the sample oriented as in Fig. 1a-c. Text indicates the number of graphene layers in various regions of the flake. Normal force setpoint was 1 nN. Scale bar: 5 μm. **b**, Simultaneously recorded friction signal, showing primarily two domains which extend over several steps in the terrace. Friction contrast between the domains does not strongly change with flake thickness. **c**, Transverse force scan of the same region, also with normal force setpoint 1 nN. Again the contrast between domains is similar for regions of different thickness. **d**, Friction signal versus normal force setpoint for regions of the flake with 1, 2, 3, and 7 layers of graphene, all within the low friction domain. The data are normalized to the friction signal on the monolayer. At low normal force, the contrast between monolayer and 7 layers is minimal, but at higher normal force the contrast grows. Error bars reflect the standard deviation of the friction signal within the sampled regions of the flake. As in Figs. 1e and 1f, the friction signal here is defined as the difference between backward scanning images and forward scanning images, divided by two, to eliminate any friction offset (see Methods).

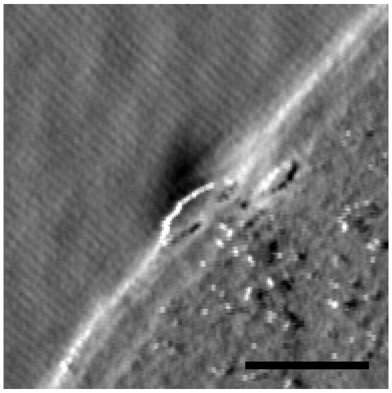

Supplementary Figure 4: Stripe-superlattice on a gold/mica substrate. Tapping-mode topography signal, differentiated along the scan axis (horizontal), at the edge of an as-deposited, few-layer graphene flake on gold on mica. Stripes are visible on the graphene (upper left half of image) with period 3.8 ± 0.2 nm; peak-to-trough amplitude in the topography signal is 20 pm. Scale bar: 50 nm.

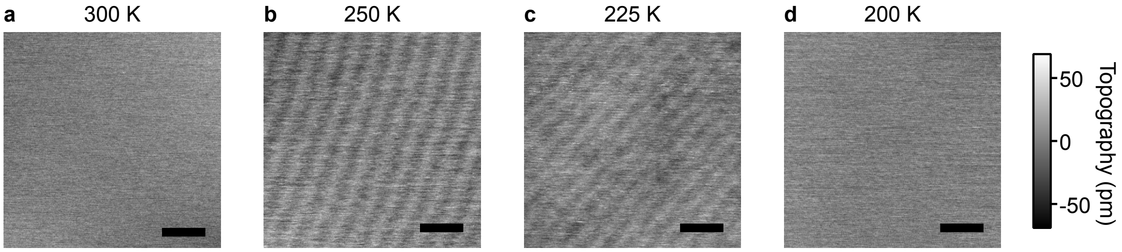

Supplementary Figure 5: Formation and suppression of stripes with decreasing temperature. **a**–**d**, Non-contact AFM topography of an hBN crystal 60 nm thick, as deposited on SiO_2, at various temperature setpoints as the temperature was lowered. Scale bars: 50 nm. At 300 K (**a**), no stripes are visible. At 250 K (**b**), stripes have formed, with peak-to-trough amplitude 15 pm. The stripes are still visible at 225 K (**c**); changes in the apparent period and stripe axis between 250 K and 225 K could result from thermal scanner drift, or from slightly differing scan locations. At 200 K (**d**), stripes can no longer be resolved. Horizontal features faintly visible in all scans, but especially in **a** and **d**, are artifacts of the horizontal scan axis.

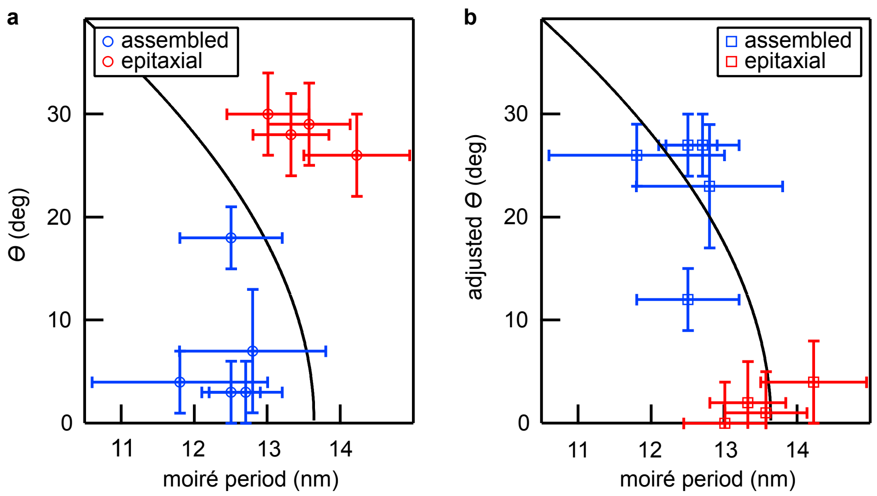

Supplementary Figure 6: Crystallographic orientation of stripe axes in graphene/hBN heterostructures. **a**, Blue circles indicate the measured rotational misalignment θ between stripe axes and nearest moiré lattice vectors for our five assembled, nearly aligned heterostructures. Red circles indicate θ for four representative epitaxial heterostructures (of 25 measured). Black curve shows the calculated angular misalignment[1] between the moiré lattice vectors and the graphene lattice vectors, assuming that the lattice constants for hBN and graphene are $a_{\mathrm{hBN}} = 0.25$ nm and $a_{\mathrm{graphene}} = a_{\mathrm{hBN}}/1.018$. Both red and blue circles should fall on this curve if the stripe axes are zigzag. **b**, Same data as in **a**, but the value plotted on the vertical axis is adjusted to be $30° - \theta$. The squares should fall on the black curve if the stripe axes are armchair. The error bars for the moiré period reflect the radial FWHM of the moiré peaks in the FFT of the tapping mode topography images. The error bars for θ account for angular uncertainty in both the stripe axes and the moiré lattice vectors, and are calculated using the quadrature sum of the angular FWHMs of the stripe-superlattice peaks and the moiré peaks in the topography FFT.

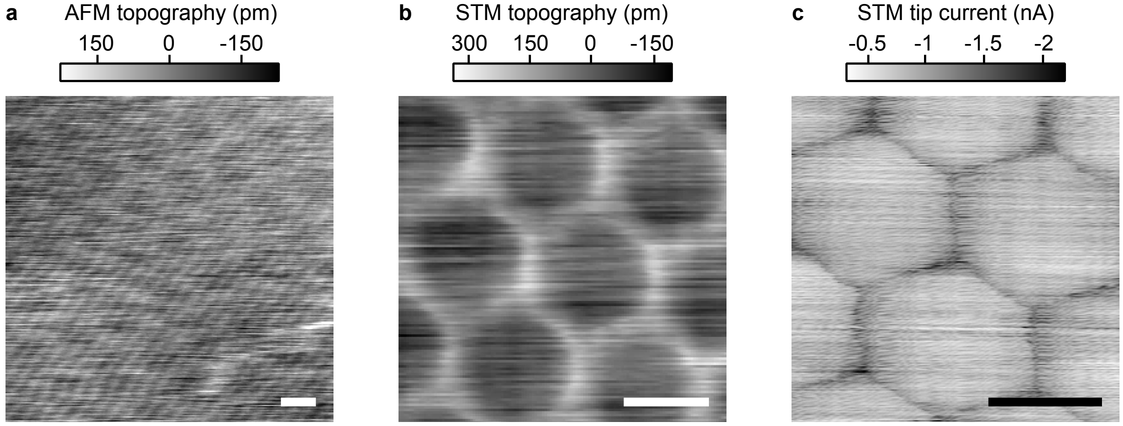

Supplementary Figure 7: Absence of stripes in STM. **a**, AFM topography of an epitaxial graphene/hBN heterostructure. The peak-to-trough topographic amplitudes of the moiré pattern and stripes are similar. Scale bar: 10 nm. **b**, STM topography of the same sample in constant current mode, with tip-sample bias 50 mV and current 1 nA. The moire pattern is clearly visible, with no evidence of the stripe-superlattice (the faint vertical stripes in this image are noise). Scale bar: 10 nm. **c**, STM topography in constant height mode, with tip-sample bias 50 mV. Again no stripes are visible, despite sharp resolution of the moiré pattern. Scale bar: 10 nm.

Supplementary Note 1: Uniformity of stripe-superlattice within a single domain

Single domains of stripes may span many microns. Here we illustrate the extreme uniformity of the stripe-superlattice by analyzing a representative large area scan (scan window approximately 600 nm) within a single domain on hBN (Supplementary Fig. 1a); results for graphene are similar. We have deliberately chosen an image that contains a phase slip to illustrate that small superlattice defects are possible—but we emphasize that phase slips are uncommon away from domain boundaries or structural defects, and that phase slips could be induced by the moving tip rather than being a feature of an unperturbed sample. The perceptible waviness of stripes on the graphene grain in the upper-left of Fig. 3a is another example of a defect. We only observe such waviness in epitaxial samples, and only near boundaries between graphene and hBN regions.

In Supplementary Fig. 1a, the peak-to-trough topographic amplitude of the stripes is 30 ± 10 pm throughout the scan window (Supplementary Fig. 1b). Angular resolution of the stripe axis is limited by slowly-varying scanner drifts, which manifest as smearing of the superlattice peak in the fast Fourier transform (FFT) along the direction perpendicular to the scan axis (Supplementary Fig. 1c). Here this smearing permitted an angular resolution of 4° (Supplementary Fig. 1d); while scan parameters can be optimized for slightly improved angular resolution (for instance, by increasing the scan speed to mitigate thermal drifts), dramatic improvement is difficult. To this few-degree precision, we observe no changes in stripe axis within a domain. The period over a large area can be measured with very high *precision*: a radial cut through the superlattice peak yields a period 6.43 ± 0.04 nm (Supplementary Fig. 1e). However, extremely slowly varing thermal drifts will cause an affine distortion of the image, which strongly impacts the *accuracy* of the measured period (and stripe axis). The large-area scan in Supplementary Fig. 1a required a particularly low scan rate, and is therefore particularly strongly affected by drift; smaller, faster scans of the same area reveal a stripe period of 4.6 ± 0.2 nm.

Supplementary Note 2: Variability in global stripe period

We typically observe a global stripe period between 4 and 5 nm for stripes on both graphene and hBN, but we sometimes observe the period to change significantly after thermal cycling a sample. For example, imaging one graphene/hBN heterostructure before (Supplementary Fig. 2a,b) and after (Supplementary Fig. 2c,d) thermal cycling between 10 K and 390 K in our PPMS revealed an increase in global stripe period of 1.6 ± 0.3 nm (here the measured change in period is very accurate, as we have used a moiré pattern in

the sample to calibrate away thermal drifts). Within the adsorbate picture, these changes in period can be explained by self-assembly of a different species following the thermal cycle, or by the addition or removal of electrolytic impurities, which modify the Debye screening length and therefore the period; for instance, changing the salt concentration was shown[2] to modify the period of self-assembled surfactants on graphite by up to 2 nm.

Supplementary Note 3: Anisotropic friction on thicker flakes

Stripes appear on both monolayer and multilayer graphene flakes, and produce domains of anisotropic friction that extend across step-edges with minor change in frictional contrast (Supplementary Fig. 3a-c). Previous work reported the friction on monolayer graphene to be twice as large as the friction on "bulk-like" flakes (4 layers or thicker), an effect ascribed to increased puckering around the tip for few-layer graphene.[3] By comparison, frictional contrast in our images (Supplementary Fig. 3b,c) is dominated by the frictional domains, and we find minimal contrast between monolayer and bulk-like flakes within the same domain.

As we increase the normal force setpoint beyond the 1 nN normal force setpoint that we typically use for imaging, contrast between monolayer and bulk-like flakes grows (Supplementary Fig. 3d), approaching agreement with the values quoted in Supplementary Ref. 3. These results are consistent with the presence of at least two distinct friction-producing effects: drag from an adsorbate layer (the stripes), which dominates at low normal force and produces friction anisotropy, and puckering of the flake, which more sharply increases with applied load and contributes isotropic friction. Supplementary Ref. 3 evidently did not observe frictional domains, despite imaging at a 1 nN normal force setpoint with probe tips nominally identical to ours (although with a different cantilever stiffness). We suspect that some aspect of the sample preparation in Supplementary Ref. 3 prevented formation of the stripe-superlattice.

We note that 4 nm stripes and anisotropic friction have been observed on HOPG by others.[4,5] Although we have not extensively studied HOPG ourselves, we have observed the stripes and anisotropic friction on exfoliated graphene flakes as thick as 50 nm, and on exfoliated hBN flakes as thick as 1 micron (these were the maximum thicknesses of graphene and hBN flakes tested; thicker flakes are often covered in tape residue). The self-assembly and friction phenomena are similar on both monolayers and these bulk-like exfoliated crystals, although we find that the frictional domains on thicker flakes are often less stable to repeated imaging.

Supplementary Note 4: Stripes on graphene on substrates other than silicon dioxide

To determine the relevance of the specific choice of substrate to the appearance of stripes, we exfoliated graphene flakes onto a variety of substrates. For graphene on 200 nm of Au(111) on mica, we observed stripes (Supplementary Fig. 4) on two flakes out of approximately ten that we checked. After thermal cycling by immersion in liquid nitrogen, we found stripes on the same two flakes, and still none on other flakes. On hardbaked SU-8 photoresist spun 15 microns thick, we found stripes on all (approximately ten) exfoliated graphene flakes that we checked. On platinum thin films (5 nm) evaporated on magnesium oxide, we found no stripes on any of approximately ten graphene flakes examined. In all cases, the stripes identified had the same approximate period and amplitude as those found on graphene on oxidized silicon substrates.

In accordance with our interpretation of the stripes as adsorbates, we hypothesized that substrate hydrophilicity might impact stripe formation on a given flake. To investigate this possibility, we measured the contact angle of water droplets (5 μL; contact area much larger than flake size) on the various substrates described above (after graphene deposition and AFM measurement), and found that the contact angle was by itself not a good predictor of the presence of stripes on the graphene flakes. For instance, both a platinum/magnesium oxide sample (no stripes on graphene) and an SU-8 sample (stripes on graphene) had a water contact angle of 80°, while the water contact angle on different SiO_2 samples with stripes ranged between 35° and 70°. The precise effect that the substrate and other environmental factors have on the self-assembly of stripes merits further investigation.

Supplementary Note 5: Variable temperature AFM measurements

Using a variable temperature AFM (VT-AFM) operating in ultrahigh vacuum (UHV; see Methods), we scanned for stripes on an as-exfoliated hBN flake on SiO_2 while holding the temperature at successively lower setpoints starting from 300 K (Supplementary Fig. 5). The sample was not baked after loading in the UHV chamber, leaving a layer of ambient adsorbates on the flake. At 300 K, we could not resolve stripes on the sample, consistent with our typical results for as-exfoliated hBN scanned under ambient conditions. Once the sample had cooled to the first low temperature setpoint (250 K), we could resolve stripes, which persisted to 225 K, but could not be resolved again at 200 K or 175 K. We stepped the temperature back up to 300 K and still could not resolve stripes at any of several temperature setpoints. We note that the images in Supplementary Fig. 5 are not corrected for thermal drift, which significantly affects the apparent angle and period of the stripes. Uncontrolled lateral movement ($\sim$1 μm) of the tip relative to the sample between

temperature setpoints also prevented us from scanning precisely the same location at every temperature on the relatively featureless hBN flakes that we studied. For these reasons, we hesitate to make any claims about the apparent change of period and stripe axis between 250 K and 225 K in Supplementary Fig. 5.

We performed similar VT-AFM experiments on two other hBN flakes (on two separate oxidized silicon pieces) which had been cycled to low temperature in a separate cryostat before loading in the VT-AFM (again not baked after loading in the UHV chamber). On both samples, we could resolve stripes at 300 K in UHV prior to decreasing the temperature. In one sample, we directly lowered the temperature from 300 K to 110 K, and found no stripes at low temperature. In the other sample, we lowered the temperature to 250 K and still observed stripes, but found them to disappear upon lowering to 200 K. Stripes did not reappear in either sample at any temperature setpoint while warming up to 300 K. Furthermore, we did not observe stripes in ambient AFM scans of either sample after removal from the VT-AFM.

Our VT-AFM study was performed at a user facility which offered limited time for our experiment. A much more extensive VT-AFM study will be required to fully understand the behavior of the stripes with temperature in UHV. We provide the following (admittedly speculative) interpretation of the limited data that we do have. The formation of stripes as the temperature is lowered from 300 K can be understood as a phase transition from an initially homogeneous layer of adsorbates: below the transition temperature, symmetry is spontaneously broken to form an ordered stripe pattern. As the temperature is further lowered, the stripe pattern disappears, which could reflect various possibilities. First, while the measured pressure in the chamber is 8×10^{-11} mbar (UHV), this pressure is not measured in the immediate vicinity of the sample, sample holder, and cantilever—all of which could still be outgassing. It is therefore possible that the stripes disappear upon further cooling because the adsorbates finally desorb from the surface, perhaps aided by cryopumping from nearby surfaces at liquid nitrogen temperature. Alternatively, if the stripes are indeed nitrogen accumulated between a water layer and the hydrophobic hBN surface,[6,7] the conditions for interfacial nitrogen enrichment may no longer hold as the water crystallizes, allowing the nitrogen to escape. The exact phase diagram of water layers depends sensitively on the host surface and the presence of coadsorbed species, but in many cases ice undergoes phase transitions around 200 K (Supplementary Ref. 8).

The fact that we no longer observe stripes even after removal from UHV to ambient may reflect hysteresis of the ordering temperature for the stripes. Assuming that the stripes are some airborne species (such as nitrogen, water, or hydrocarbons), removing the sample from UHV to ambient will readsorb the relevant species, but the adsorbates may not order at 300 K. On the other hand, if the relevant species are adsorbed below the ordering temperature, stripes could form at low temperature and persist to 300 K if the ordering temperature is hysteretic. In agreement with this hysteresis picture, we almost always observed stripes at

300 K following thermal cycles in poorer vacuum conditions than UHV (Methods).

Supplementary Note 6: Crystallographic orientation of stripes

For heterostructures with close alignment between graphene and hBN (including some assembled heterostructures, and all epitaxial heterostructures), we extract the crystallographic orientation of the stripe axes using the calculated angular misalignment[1] between the moiré lattice vectors and the graphene lattice vectors. Our data rule out the possibility that the stripe axes are parallel to the graphene lattice vectors (zigzag axes), since the measured angle between the stripe axes and the nearest moiré lattice vector does not follow the expected trend as a function of moiré superlattice period (Supplementary Fig. 6a). In contrast, our angular misalignment data conform to expectations if we assume that the stripe axes are always armchair (Supplementary Fig. 6b).

Supplementary Note 7: Absence of stripes in STM

To help distinguish between explanations for the stripes, we studied epitaxial graphene on hBN under ambient conditions by both AFM and STM, with the STM tunneling parameters optimized for resolution of the atomic lattice and moiré pattern. The tapping mode AFM scans reveal stripes of topographic amplitude comparable to the moiré corrugation, but the STM scans in both constant current and constant height modes only reveal a moiré pattern with no stripes (Supplementary Fig. 7). These data are consistent with self-assembled stripes of adsorbates: the stripes would be disturbed by the STM tip, which sits angstroms from the graphene surface when imaging the graphene lattice. If the stripes were structural ripples, we would expect to see them in both AFM and STM topography.

Supplementary Note 8: Detecting adsorbates in tapping and contact modes

In tapping mode, a fixed drive power is applied at a frequency near the resonant frequency of the cantilever, causing the tip mounted on the cantilever's free end to oscillate. As the cantilever and oscillating tip are brought toward a surface, the tip starts to feel forces from the sample surface. These forces shift the amplitude and phase of oscillation, which in our experiment are measured optically. A feedback loop tries to maintain constant oscillation amplitude by adjusting the cantilever's vertical position. The vertical position is then mapped as a function of lateral position, and interpreted as the topography signal.

But the equivalence between the vertical position and sample topography only holds for a chemically homogeneous surface. If the tip interacts with a layer of adsorbates, the oscillation amplitude and phase will in general shift differently for different adsorbed species. The target amplitude for the feedback loop can therefore be chosen so that different species appear with different heights, even if the adsorbed layer is uniformly thick and the underlying solid surface is flat. In our experiments, we set the target amplitude so that the topography signal detects the species that orders into stripes. The measured height of the stripes in the topography signal does not necessarily reflect the "true" height of the adsorbed species, and indeed we find that the measured height depends on our tip conditions and target amplitude.

We believe that the stripes are quite firm compared to any water layer or other adsorbates present, and that our AFM tip therefore does not substantially penetrate or disturb the stripes in tapping mode, making the stripes easy to detect. But even if the tip does displace the stripes, they might still be detectable. The tip oscillation amplitude is tens of nanometers, while the stripes are are at most a few nanometers thick, so the tip penetrates the stripes for at most a small fraction of an oscillation period. The stripes may have ample time to reconstruct between successive interactions with the tip.

In contrast, with small oscillation amplitude and small tip-sample distance, visibility of stripes can be entirely suppressed in AFM images. Wastl *et al.*[9] studied striped adsorbates apparently identical to ours, but on epitaxial multilayer graphene, using an AFM tip mounted on a quartz tuning fork with an oscillation amplitude of 0.2 nm. When the tip was held sufficiently far away from the graphene surface, the stripes could be observed. When the tip was brought close enough to atomically resolve the graphene lattice by sensing short-range forces, the stripe structure completely disappeared, as the constant proximity of the tip to the surface prevented the stripes from forming near the tip.

In our contact mode measurements, the tip is scanned laterally while maintaining contact with the surface. The cantilever twists while scanning as a result of in-plane drag forces felt by the tip; this twist is optically detected and interpreted as the friction signal. If we assume that the tip pushes through the adsorbates and stays in contact with the underlying graphene or hBN, the tip must then kick the adsorbates out of its way as it moves along the surface. The reaction force from these in-plane kicks creates a friction signal due to the adsorbates.

Most of our friction images are taken with a 1 nN normal force. At 1 nN, we measure monolayer graphene sheets to be artificially thick (usually about 1 nm instead of the "true" thickness of 0.3 nm), suggesting that an adsorbate layer is still present between tip and graphene. When we apply more than $\sim$10 nN, we recover the expected thickness of 0.3 nm. It is possible that at low normal force, the tip does not disturb the stripe structure; we expect friction anisotropy due to the adsorbates either way.

Supplementary References

[1] Tang, S. *et al.* Precisely aligned graphene grown on hexagonal boron nitride by catalyst free chemical vapor deposition. *Sci. Rep.* **3**, 2666 (2013).

[2] Wanless, E. J. & Ducker, W. A. Organization of sodium dodecyl sulfate at the graphite-solution interface. *J. Phys. Chem.* **100**, 3207–3214 (1996).

[3] Lee, C. *et al.* Frictional characteristics of atomically thin sheets. *Science* **328**, 76–80 (2010).

[4] Rastei, M. V., Heinrich, B. & Gallani, J. L. Puckering stick-slip friction induced by a sliding nanoscale contact. *Phys. Rev. Lett.* **111**, 084301 (2013).

[5] Rastei, M. V., Guzmán, P. & Gallani, J. L. Sliding speed-induced nanoscale friction mosaicity at the graphite surface. *Phys. Rev. B* **90**, 041409 (2014).

[6] Lu, Y.-H., Yang, C.-W. & Hwang, I.-S. Molecular layer of gaslike domains at a hydrophobic water interface observed by frequency-modulation atomic force microscopy. *Langmuir* **28**, 12691–12695 (2012).

[7] Wastl, D. S. *et al.* Observation of 4 nm pitch stripe domains formed by exposing graphene to ambient air. *ACS Nano* **7**, 10032–10037 (2013).

[8] Henderson, M. A. The interaction of water with solid surfaces: fundamental aspects revisited. *Surf. Sci. Rep.* **46**, 1–308 (2002).

[9] Wastl, D. S., Weymouth, A. J. & Giessibl, F. J. Atomically resolved graphitic surfaces in air by atomic force microscopy. *ACS Nano* **8**, 5233–5239 (2014).
